# Cancer-Secreted Exosomal MiR-620 Inhibits ESCC Aerobic Glycolysis *via* FOXM1/HER2 Pathway and Promotes Metastasis

**DOI:** 10.3389/fonc.2022.756109

**Published:** 2022-05-16

**Authors:** Yanbo Zhu, Fang Li, Yilong Wan, Hansi Liang, Si Li, Bo Peng, Liqun Shao, Yunyun Xu, Dong Jiang

**Affiliations:** ^1^ Department of Oncology, First Affiliated Hospital of Soochow University, Suzhou, China; ^2^ Department of Human Anatomy and Histology & Embryology, The School of Biology & Basic Medical Sciences, Soochow University, Suzhou, China; ^3^ Department of Thoracic Surgery, The First Affiliated Hospital of Soochow University, Suzhou, China; ^4^ Institute of Thoracic Surgery, The First Affiliated Hospital of Soochow University, Suzhou, China; ^5^ Jiangsu Institute of Clinical Immunology, Jiangsu Key Laboratory of Gastrointestinal Tumor Immunology, The First Affiliated Hospital of Soochow University, Suzhou, China; ^6^ Clinical Medicine Major, Soochow University Medical College, Suzhou, China; ^7^ Childrens’ Hospital Affiliated to Soochow University, Institute of Pediatrics, Suzhou, China

**Keywords:** miR-620, exosomes, esophageal squamous cell carcinoma, aerobic glycolysis, metastasis

## Abstract

**Background:**

Esophageal squamous cell carcinoma (ESCC) is a leading cause of cancer death worldwide. MicroRNAs (MiRNAs) have been reported to regulate cell functions through exosomes. Through the Gene Expression Omnibus (GEO) database, miR-620 was selected as a serum miRNA highly expressed in ESCC, but its detailed role in ESCC has not been explored. Tumor-secreted miRNAs have been reported to promote cancer metastasis through reprogramming the aerobic glycolysis of lung fibroblasts. Therefore, we intended to verify whether exosomal miR-620 secreted in ESCC cells may regulate the aerobic glycolysis of lung fibroblasts.

**Methods:**

The effect of miR-620 on the aerobic glycolysis of ESCC cells was firstly verified through bioinformatics prediction and mechanism assays. Exosomes secreted from ESCC cells was detected, and the influence of exosomal miR-620 in regulating the aerobic glycolysis of lung fibroblasts was then verified both *in vitro* and *in vivo*.

**Results:**

MiR-620 inhibited ESCC malignancy and suppressed the aerobic glycolysis of ESCC cells *via* targeting Forkhead box M1 (FOXM1) and human epidermal growth factor receptor 2 (HER2). Moreover, exosomal miR-620 was highly secreted in ESCC and could regulate HFL1 aerobic glycolysis *via* FOXM1/HER2 signaling. Furthermore, exosomal miR-620 could promote ESCC metastasis by reprogramming the aerobic glycolysis of lung fibroblasts (HFL1).

**Conclusion:**

Exosomal miR-620 secreted by ESCC cells inhibited the aerobic glycolysis *via* FOXM1/HER2 axis and promoted cancer metastasis.

## Introduction

Esophageal cancer is one of the leading causes of cancer-related death worldwide, and half of the cases of the disease occur in China (1). As one of the predominant histological subtypes of esophageal cancer, esophageal squamous cell carcinoma (ESCC) is characterized by late-stage diagnosis, metastasis, therapy resistance, and frequent recurrence, which results in challenging clinical management and effective therapeutic options (2). Therefore, it is urgently needed to explore more targeted biomarkers for ESCC treatment.

MicroRNAs (miRNAs) are small noncoding RNAs of 22 nucleotides, which have increasingly been recognized as potent posttranscriptional regulators of gene expression (3). They have been reported to function posttranscriptionally by usually base-pairing to the mRNA 3′-untranslated regions (3′UTR) (4). Exosomes, which are one of the smallest extracellular vesicles released from cells, have been shown to carry different nucleic acids, including miRNAs (5, 6). More and more evidences have demonstrated that miRNAs can regulate cell functions not only between cells of the same origin but also to distant target cells (7, 8). In this study, we applied the GEO database (https://www.ncbi.nlm.nih.gov/geo/) (id: GSE122497) to obtain the serum microRNA profile of ESCC samples. Among them, many miRNAs have been demonstrated to play important parts in cancer development, especially in ESCC. Genes regulated by antitumor miR−145−3p, for example, were closely associated with the molecular pathogenesis of ESCC (9); miR-10a, miR-22, miR-100, miR-148b, miR-223, miR-133a, and miR-127-3p have been discovered to be with significant high expression in the serum of patients with ESCC (10). Combined with relevant literature, we further searched for the miRNAs that have been reported to be associated with aerobic glycolysis, and finally a serum miRNA-miR-620 was sifted out as our study subject. MiR-620 has been reported to participate in cancer progression. For example, miR-620 promotes TGF-β1-induced proliferation of airway smooth muscle cell (11), participates in the regulation of cervical cancer cell proliferation, invasion and migration (12) and facilitates the resistance of triple negative breast cancer cells to gemcitabine treatment (13). What is more, exosomal miR-620 derived from tumor has been demonstrated as a promising diagnostic and prognostic noninvasive biomarker in nonsmall-cell lung cancer patients (14). However, the role of miR-620 in ESCC cells and its relation with exosomes in regulating ESCC progression remain unclear.

Reprogrammed energy metabolism to fuel rapid cell growth and proliferation is an emerging hallmark of cancer, and reprogramming of energy metabolism to promote rapid cell growth and proliferation is an emerging feature of cancer (15). In most cancers, even when oxygen is sufficient, cancer cells can promote nutrient uptake and absorption through aerobic glycolysis and reducing mitochondrial oxidative phosphorylation, which is known as the Warburg effect (16, 17). Aerobic glycolysis is the process of oxidation of glucose into pyruvate followed by lactate production under normoxic condition, and it is one of the earliest known evidences of metabolic alteration in neoplasms (18).

Related document has reported the role of exosomal miRNAs in cancer-associated fibroblasts (19). Breast cancer-secreted miR-122 reprograms lung fibroblast aerobic glycolysis and thus promoting metastasis (20). Also, it was discovered that cancer cell-secreted IGF2 instigates fibroblasts and bone marrow-derived vascular progenitor cells to promote cancer progression (21). However, related research in ESCC has not been documented, which leads to our study. In this study, lung fibroblast HFL1 was cocultured with exosomal miR-620 to construct HFL1/Exo cells, and a series of mechanism experiments were conducted to explore the impact of exomal miR-620 on lung fibroblast aerobic glycolysis and further on the progression of ESCC.

In a word, we aim to verify the upregulation expression of miR-620 in ESCC cells and further explored the effects of miR-620 on lung fibroblast aerobic glycolysis in ESCC.

## Materials and Methods

### Ethical Statement

Relevant animal experiments were carried out in accordance with the approval of the ethics committee of the First Affiliated Hospital of Soochow University. All experiments are taken in accordance with the standard biosecurity and safety procedures of the First Affiliated Hospital of Soochow University.

### Cell Lines

Human normal esophageal epithelial Het-1A cell line and human ESCC cell lines (ECA-109 and KYSE450) were obtained from American Type Culture Collection (ATCC, Manassas, VA, USA). The luciferase-labeled ECA-109-luc and KYSE450-luc cells were obtained from Shanghai Chenyishiye Co., Ltd (Shanghai, China). The above cells were cultivated in Dulbecco’s modified Eagle’s medium (DMEM) (MD207-050, Gibco-BRL/Invitrogen, Waltham, MA, USA) supplemented with 10% fetal bovine serum (FBS; 16000-044, Gibco, USA) and 100× penicillin/streptomycin solution (15140122, Gibco-BRL/Invitrogen, USA). Human embryonic lung fibroblast HFL1 cell line was obtained from BeNa Culture Collection (China) and cultured with 90% F-12K plus 10% FBS.

### Real-Time Quantitative RT-PCR

In line with the instruction of TRIzol reagent (T9108, Takara, Japan), the isolation of total RNA samples was extracted in ECA-109 and KYSE450 cells. Synthesis of complementary DNA (cDNA) for miRNAs was carried out using TaqMan™ MicroRNA Reverse Transcription Kit (TaKaRa, Kusatsu, Japan), and the cDNA for mRNAs was synthesized by PrimeScript RT reagent Kit (TaKaRa, Japan). RT-qPCR reaction was achieved with qRT-PCR Kit (QR0100-1KT, Sigma-Aldrich, St. Louis, MO, USA) followed by 2^−ΔΔCt^ method. In relevant assays, the reference gene was β-actin while the reference gene for miRNA quantification was U6 and miR-16 (in exosome experiments). Related sequence information is included in **Supplementary Table S1**.

### Cell Transfection

Specific shRNAs targeting FOXM1 (sh1/2/3-FOXM1) and HER2 (sh1/2/3-HER2), together with their negative control shRNA (sh-NC) were provided by RiboBio Co., Ltd. (Guangzhou, China) to silence FOXM1 expression. For the overexpression of FOXM1 and HER2, the whole sequences were synthesized and subcloned into pcDNA3.1 vector to construct pcDNA3.1-FOXM1 and pcDNA3.1-HER2, with pcDNA3.1 empty vector as the negative control (NC). For the overexpression or silencing of miR-620, miR-620 mimics or miR-620 inhibitor was respectively used, with mimics-NC or inhibitor-NC as internal controls. Transfections were conducted with Lipofectamine 2000 (11668019, Invitrogen, USA) in line with the supplier’s protocols. After incubation for 48 h, cells were collected for subsequent experiments.

### Cell Counting Kit-8 Assay

Cell viability was evaluated with the application of Cell Counting Kit-8 (CCK-8) according to the manufacturer’s guidelines. Transfected ECA-109 or KYSE450 cells were seeded to 96-well plates and then placed in an incubator with 5% CO_2_ at 37°C for 24 h. Next, 10 μl of CCK-8 solution (Dojindo, Kumamoto, Japan) was added into each pore of the plate for 1-h incubation. Finally, the absorbance at 450 nm after 24, 48, or 72 h was measured with the microplate reader to evaluate the viability of indicated cells.

### Wound-Healing Assay

ECA-109 and KYSE450 cells were seeded into the 6-well plates and then cultivated at 37°C with 5% CO_2_ for confluence. When the degree of cell confluence was above 90%, a pipet tip was used to scrape the cells as vertically as possible and then the cells were washed with PBS for three times. The scratched cells were removed, the serum-free medium was added, and then the cells were placed into an incubator at 37°C with 5% CO_2_. Finally, the scratch was imaged by microscope at 0 and 24 h for analysis.

### Transwell Assays

ESCC cells were planted on the top of 24-well Transwell chambers coated with Matrigel for invasion assay or without Matrigel for migration assay. The lower chambers were loaded with complete medium. Twenty-four hours later, cells in the upper layer were removed by a cotton swab and the bottom of the chamber was fixed in methanol solution for 15 min. Crystal violet was adopted to stain the membranes for 10 min, and the invaded or migrated cells were observed and counted under a microscope (10 × 10).

### Western Blot Assay

When the cell confluence was above 80%, the protein extracts were collected from ESCC cell lines using protein extraction kit (PROTTOT-1KT, Sigma-Aldrich, USA) and RIPA buffer (KGP701, KeyGEN BioTECH, Nanjing, China). After being separated through sodium dodecyl sulfate polyacrylamide gel electrophoresis (SDS-PAGE; P0670-250ml, Beyotime Biotech, Shanghai, China), proteins were transferred to polyvinylidene fluoride (PVDF) membranes and cultured in 5% skim milk. The membranes were cultivated with primary antibodies over night at 4°C, followed by being cultivated with secondary antibody for 1 h. After washing in TBST, the secondary antibodies were added and finally assayed by ECL substrate.

The primary antibodies were obtained from Abcam (UK) and listed as follows: anti-E-cadherin (ab40772), anti-Vimentin (ab92547), anti-CD63 (ab1318), anti-CD81, anti-HSP70 and anti-FOXM1 (ab245309), anti-GM130, with anti-β-Actin (ab8227) as the internal control. Experiment was conducted three times.

### RNA Pull-Down Assay

With the application of Pierce™ Streptavidin Magnetic Beads (Thermo Fisher, USA), RNA pull-down assay was carried out in ECA-109 cells. Experimental groups were divided into Input Antisense (control), HER2 Promoter Sense, and HER2 Promoter (mut) Sense groups. Biotinylated HER2 probes were incubated with cell extracts and streptavidin magnetic beads and then RNAs were purified with TRIzol reagent. The enrichment of FOXM1 was analyzed by Western blot. Likewise, biotinylated FOXM1 probes were incubated with cell extracts and streptavidin magnetic beads, and, finally, the enrichment of miR-620 was examined by qRT-PCR. The experiment was independently conducted in triplicate.

### Luciferase Reporter Assay

For luciferase reporter assay, FOXM1 3′UTR possessing wild-type and mutant miR-620 binding sites were subcloned into pmirGLO luciferase vectors to obtain pmirGLO-FOXM1 (3′UTR) and pmirGLO-FOXM1 (3′UTR-Mut). MiR-620 mimics or mimics NC were cotransfected with pmirGLO-FOXM1 (3′UTR) and pmirGLO-FOXM1 (3′UTR-Mut) into NSCLC cells. After 48-h transfection, cells were extracted and the luciferase activities were analyzed utilizing the luciferase reporter assay system (Promega).

As for FOXM1 and HER2 promoter luciferase reporter assay, the HER2 promoter was constructed into pGL3 luciferase vectors and then cotransfected with pcDNA3.1 or pcDNA3.1-FOXM1 into 293T cells. The luciferase activities were analyzed utilizing the luciferase reporter assay system (Promega).

### Exosome Uptake Experiment

By referring to related document, we conducted the exosome uptake experiment (22). A total of 20 ml of culture media (1 × 10 (7) cells) were collected on ice, centrifuged at 800×*g* for 10 min, and then centrifuged at 12,000×*g* for 30 min to remove the cellular debris. Exosomes were centrifuged at 100,000×*g* for 2 h in a SW32 rotor (Beckman Coulter) for separating from the supernatant. The exosome pellet was washed once in a large volume of PBS and then resuspended in 100 μl of PBS. Finally, exosomes were then identified by transmission electron microscope (TEM) to observe the morphologies of exosomes secreted from miR-620 (labeled as miR-620-Exo).

In addition, the exosomes were marked by PKH67 Fluorescent Cell linker kits according to previous guidance (23). Briefly, ECA-109 and KYSE450 cells were seeded into 24-well plates and incubated at 37°C, with 5% CO_2_. The exosomes marked by PKH67 as well as ESCC cells were cocultured without light for 12 h and washed with PBS for three times and then fixed by paraformaldehyde for 20–30 min, rinsed by PBS for three times; the nuclei were stained by DAPI for 5 min, rinsed by PBS for three times, and fixed. Finally, the distribution of fluorescence was observed by a laser scanning microscope.

### Relevant *In Vivo* Assays

As the literature on the construction of orthotopic esophageal xenograft tumor has not been retrieved, we referred to the construction method of orthotopic breast xenograft tumor and conducted the following experiment 18. NSG mice was injected intracardiac with 2×105 luciferase-labelled KYSE450-luc cells combined with Matrigel (BD Biosciences; San Jose, CA) in a 1:1 ratio. Starting from day 3 after cancer cell transplantation, oligos (25 mg/kg) were intraperitoneally (i.p.) injected daily for 5 days and then twice weekly until the end of experiment. The mice were sacrificed after four weeks and tumour volume (mm3) was assessed by calliper measurements using the formula (width2 × length)/2.

To verify the effect of exosomal miR-620 on the aerobic glycolysis of HFL1 cells, *in vivo* experiments were conducted as previously described (20).

The female mice (6–8-week-old) were acquired commercially from the Institute of Zoology, Nanjing University. All the relevant animal experiments were carried out in accordance with the Animal Care and Use Committee guidelines of the First Affiliated Hospital of Soochow University. We intravenously injected exosomes containing low or high levels of miR-620 into mice and measured the aerobic glycolysis change in MLF. Female NOD/SCID/IL2Rγ-null (NSG) mice aged 6–8 weeks were used in this study. Exosomes were isolated from ECA-109 and KYSE450 cells, resuspended in PBS, and followed by centrifugation at 16,000×*g* for 10 min at 4°C. The supernatant was then transferred to a new tube for mouse injection biweekly for 3.5 weeks (~6 μg/injection).

### Statistical Analysis

All data from experiments including three biological replications were exhibited as the mean ± standard deviation (SD). Data analysis was achieved by Student’s *t*-test (comparison for two groups) and one-way/two-way ANOVA (comparison for more than two groups), applying SPSS 19.0 software (IBM SPSS, Armonk, NY, USA). The statistical significance in differences were confirmed when *p* < 0.05.

## Results

### MiR-620 Inhibits ESCC Cell Proliferation, Migration, Invasion, and EMT Process

**Figure 1:**
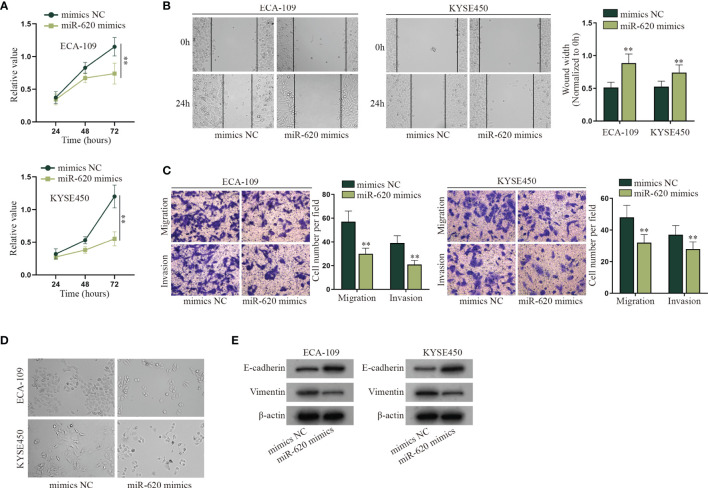
MiR-620 inhibits cell proliferation, migration, invasion, and EMT process in ESCC. **(A)** CCK-8 assay was taken to detect the proliferative ability of ESCC cells after miR-620 was overexpressed. **(B)** Wound-healing assay was conducted to detect the migration of the ESCC cells transfected with miR-620 mimics. **(C)** Transwell assay was carried out to detect the migratory and invasive ability of ESCC cells upon miR-620 overexpression. **(D)** The phenotype of ESCC cells upon miR-620 overexpression was observed through a microscope. **(E)** Western blot was utilized to detect the expression of the EMT-related proteins (E-cadherin and Vimentin) upon miR-620 overexpression. ^**^ *p* < 0.01.

With the application of the GEO database (GSE122497), serum microRNA profiles of 5,531 samples which consist of 566 esophageal squamous cell carcinoma and 4,965 of noncancer controls were exhibited. To make further selection, we searched for relevant literature to find the miRNAs associated with aerobic glycolysis. Finally, a serum miRNA named miR-620 was sifted out (**Supplementary Figure S1A**). It has been proven to promote cancer development, but how it may function in ESCC has not been identified. The role of miR-620 has been revealed in various cancers, but how it may function in ESCC has not been identified. It was observed through CCK-8 assay that the viability of ECA-109 and KYSE450 cells was suppressed by miR-620 overexpression (**Figure 1A**). Next, it was shown from wound-healing assay that the wound width was increased upon miR-620 overexpression, which indicated that miR-620 overexpression inhibited the proliferation of ESCC cells (**Figure 1B**). Results of Transwell assay manifested that the enhanced miR-620 expression inhibited the migration and invasion of ECA-109 and KYSE450 cells (**Figure 1C**), and fewer spindle-shaped ESCC cells were observed through microscope upon miR-620 overexpression (**Figure 1D**). Finally, it was seen from Western blot that after miR-620 was upregulated in ECA-109 and KYSE450 cells, the protein expression of E-cadherin enhanced while that of Vimentin declined, indicating that miR-620 overexpression suppressed the EMT process in ESCC (**Figure 1E**). The above data demonstrated that miR-620 suppressed the malignant progression of ESCC cells. Furthermore, we designed the interference sequence of miR-620 and conducted related assays to verify the influence of miR-620 silencing on ESCC cell progression. As shown in **Supplementary Figures S1B–F**, decreased miR-620 expression accelerated the proliferation, migration, invasion, and EMT process of ESCC cells, which further manifested the oncogenic property of miR-620 in ESCC.

### MiR-620 Inhibits the Aerobic Glycolysis of ESCC Cells

**Figure 2:**
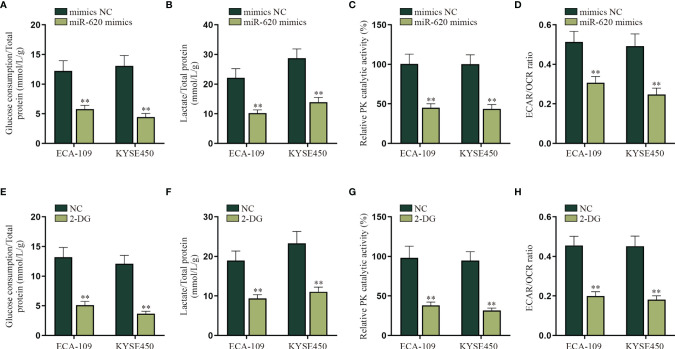
MiR-620 inhibits the aerobic glycolysis of ESCC cells. **(A)** Glucose determination kit was applied to examine the glucose determination of ESCC cells transfected with miR-620 mimics. **(B)** Lactic acid determination kit was used to detect lactic acid content in ESCC cells upon miR-620 overexpression. **(C)** Pyruvate kinase activity assay kit was applied to measure the PK content in miR-620 mimic-transfected ESCC cells. **(D)** The ECAR/OCR ratio in miR-620 mimic-transfected ESCC cells was tested by Seahorese XF Extracellular Flux Analyzer. **(E–H)** ESCC cells were treated with 50 or 100 μM 2-DG (the glycolytic inhibitor) for 48 h, and the glucose consumption, total lactate protein, relative PK catalytic activity, and the ECAR/OCR ratio were respectively examined *via* qRT-PCR. ^**^ *p* < 0.01.

Energy metabolism reprogramming has become an emerging hotspot in cancer research. In most of the cancers, even with abundant oxygen, cancer cells will promote the uptake and absorption of nutrients needed by newborn cells through aerobic glycolysis and reduce oxidative phosphorylation of mitochondria (16). Combining with the information above, we decided to explore whether miR-620 may regulate the aerobic glycolysis of ESCC cells. With the application of glucose determination kit, lactic acid determination kit and pyruvate kinase activity assay kit, it was verified that the glucose consumption, total lactate protein, and relative PK catalytic activity were reduced by miR-620 overexpression (**Figures 2A** **–C**). It was then measured through Seahorse XF Extracellular Flux Analyzers that the ECAR/OCR ratio was declined in ESCC cells transfected with miR-620 mimics (**Figure 2D**). By comparison, we analyzed the effect of miR-620 knockdown on the aerobic glycolysis of ESCC cells, and opposite results were exhibited (**Supplementary Figures S2A–D**). The above findings suggested that miR-620 inhibited the aerobic glycolysis of ESCC cells. For further verification, we treated ESCC cells with 50 or 100 μM 2-DG (the glycolytic inhibitor) for 48 h (24), and it was then discovered that the glucose consumption, total lactate protein, relative PK catalytic activity, and the ECAR/OCR ratio were all reduced upon 2-DG treatment (**Figures 2E** **–H**), which demonstrated the effective inhibition of 2-DG on ESCC aerobic glycolysis. Moreover, **Supplementary Figures S2E–H** shows that adding 2-DG could reverse the promoted aerobic glycolysis caused by miR-620 inhibitor, which further manifested that miR-620 regulated the aerobic glycolysis of ESCC cells.

### MiR-620 Regulates HER2 Expression Through FOXM1

**Figure 3:**
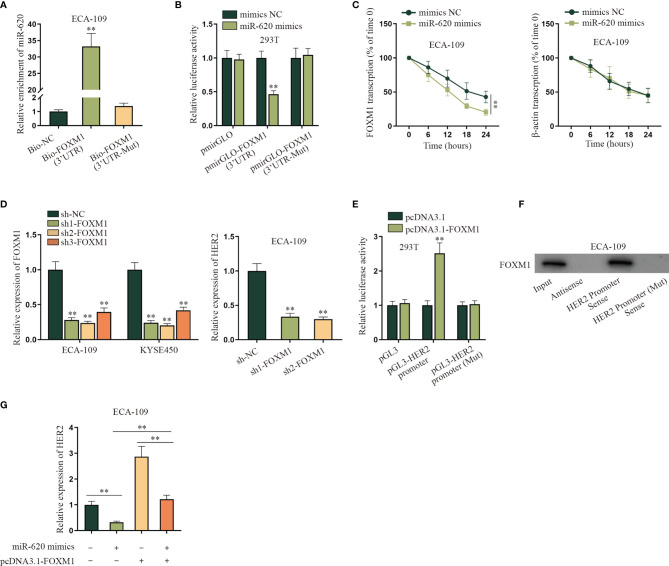
MiR-620 regulates HER2 expression through FOXM1. **(A)** RNA pull-down assay with qRT-PCR was adopted to detect the enrichment of miR-620 in Bio-FOXM1 (3′UTR) group in ECA-109 cells. **(B)** Luciferase reporter assay was conducted to test the luciferase activity of HEK293T cells transfected with miR-620 mimics in pmirGLO-FOXM1 (3′UTR) group. **(C)** A total of 50 mM α-amanitin was used to treat ECA-109 cells, and qRT-PCR was used to examine the stability of FOXM1 mRNA upon miR-620 upregulation. **(D)** FOXM1 expression was reduced in ECA-109 cells, and the expression of HER2 upon FOXM1 inhibition was tested by qRT-PCR. **(E)** Luciferase reporter assay was conducted to detect the luciferase activity of HEK293T cells transfected with pcDNA3.1-FOXM1 in pGL3-HER2 promoter group. **(F)** RNA pull-down assay with Western blot was carried out to evaluate the enrichment of FOXM1 pulled down by HER2 promoter antibody. **(G)** qRT-PCR was used to examine the expression of HER2 in ECA-109 cells upon different transfection treatments. ^**^ *p* < 0.01.

After we confirmed that miR-620 could regulate the aerobic glycolysis of ESCC cells, we continued to probe into the specific molecular mechanism. Through the GEO database (GSE75241), we selected the top 30 genes upregulated in ESCC, and then potential mRNAs which could bind to miR-620 were selected through starBase database. As a result, 8 candidates were sifted out, which were ITM2C, FNDC38, CD276, LAMC2, LHFPL2, LAMC1, PARVB, and FOXM1 (**Supplementary Figures S3A, B** **)**. Through qRT-PCR detection, it was found that only FOXM1 was significantly reduced by miR-620 overexpression in ECA-109 cells (**Supplementary Figure S3C**). Therefore, we chose FOXM1 for further investigations. The result of RNA pulldown assay with qRT-PCR showed that the enrichment of miR-620 was enhanced in the wild-type Bio-FOXM1 (3′UTR) group rather than in the mutant group (**Figure 3A**). Subsequently, it was observed through luciferase reporter assay that the luciferase activity of HEK293T cells transfected with miR-620 mimics was declined in the pmirGLO-FOXM1 (3′UTR) group while on obvious change was seen in the corresponding mutant group (**Figure 3B**). We utilized 50 mM α-amanitin to treat ESCC cells, and then it was discovered that the upregulation of miR-620 inhibited the stability of FOXM1 mRNA in ECA-109 cells (**Figure 3C**). The above findings manifested that miR-620 combined with FOXM1 3′UTR region to decline FOXM1 mRNA stability in ESCC cells. In order to figure out the downstream target gene of FOXM1, KEGG (https://www.genome.jp/kegg/) was applied and potential 6 genes related to aerobic glycolysis were detected (**Supplementary Figure S3D**), and then it was verified through hTFtarget database (http://bioinfo.life.hust.edu.cn/hTFtarget#!/) that only HER2 was forecast to be the target gene of FOXM1 (**Supplementary Figure S3E**). Therefore, HER2 was chosen for the next-step researches. It was shown from qRT-PCR assay that HER2 expression was suppressed upon sh1/2-FOXM1 transfection in ECA-109 cells, as evidenced by qRT-PCR assay (**Figure 3D**). In addition, FOXM1 expression was enhanced by transfecting pcDNA3.1-FOXM1 in ESCC cells (**Supplementary Figure S4A**), and then the result of luciferase reporter assay manifested that HEK293T cells displayed an elevated luciferase activity in pGL3-HER2 promoter cotransfected with pcDNA3.1-FOXM1, while the corresponding mutant group exhibited no obvious change (**Figure 3E**). Through RNA pulldown assay with Western blot, it was shown that FOXM1 was pulled down by the HER2 promoter sense (**Figure 3F**). Finally, qRT-PCR data showed that the addition of pcDNA3.1-FOXM1 could normalize the reduced HER2 expression caused by miR-620 overexpression in ECA-109 cells (**Figure 3G**). To conclude, FOXM1 combined with the HER2 promoter so as to transcriptionally activate HER2 expression in ESCC cells.

### FOXM1 Promotes the Aerobic Glycolysis of ESCC Cells *via* Upregulating HER2

**Figure 4:**
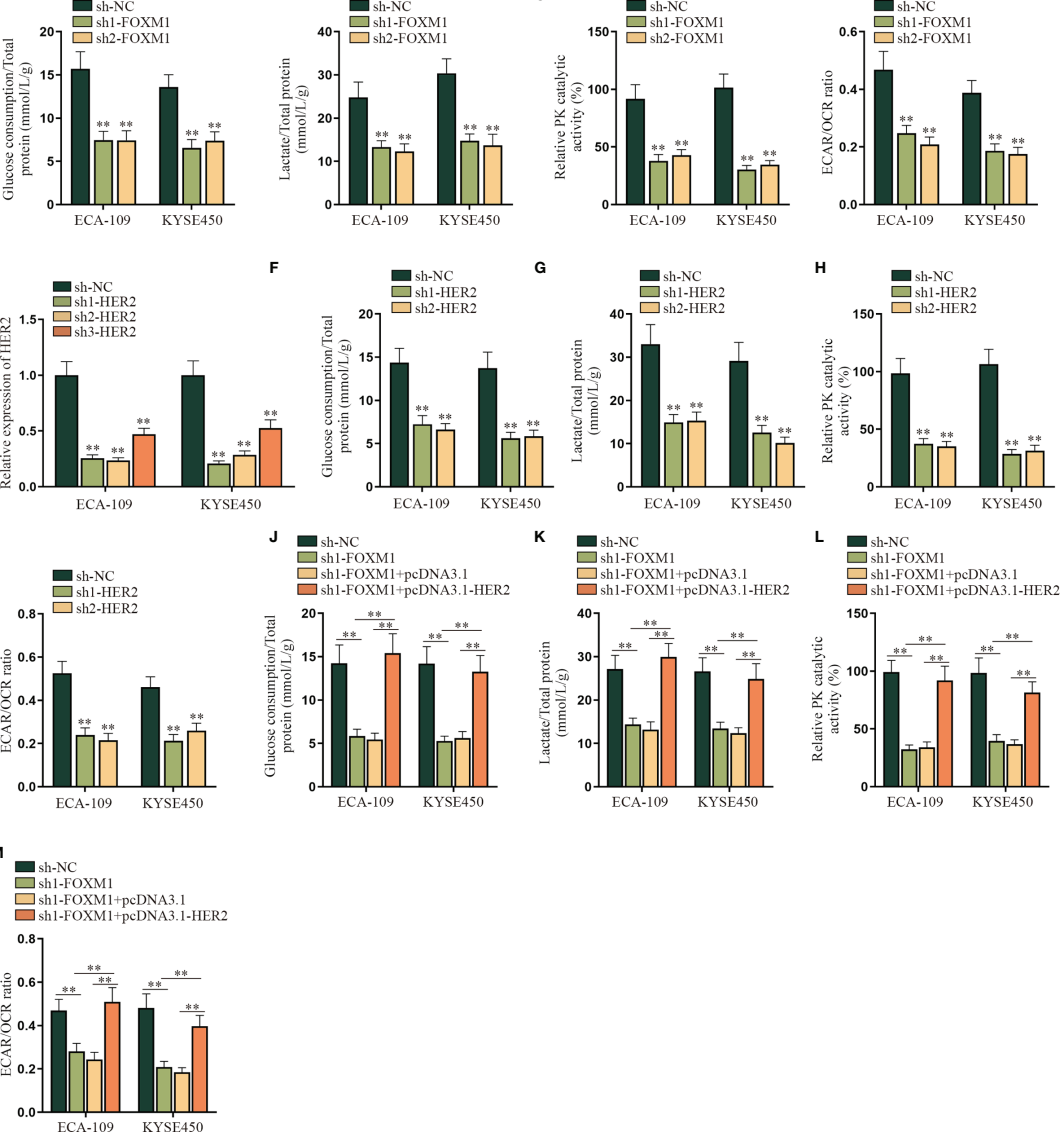
FOXM1 promotes the aerobic glycolysis of ESCC cells *via* up-regulating HER2. **(A–I)** The impact of sh-FOXM1 and sh-HER2 transfection on the aerobic glycolysis of ESCC cells was respectively evaluated including the measurement of glucose consumption, total lactate protein, relative PK catalytic activity, and the ECAR/OCR ratio. **(J–M)** Experimental groups were divided into sh-NC, sh1-FOXM1, sh1-FOXM1+pcDNA3.1, and sh1-FOXM1+pcDNA3.1-HER2 groups, and glucose consumption, total lactate protein, relative PK catalytic activity, and the ECAR/OCR ratio were respectively examined by qRT-PCR in different groups. ^**^ *p* < 0.01.

In this part, we tried to verify the regulatory mechanism of FOXM1 and HER2 on the aerobic glycolysis of ESCC cells. As shown in **Figure 4A–D**, the aerobic glycolysis of ESCC cells was suppressed after FOXM1 was knocked down, as the glucose consumption, total lactate protein, relative PK catalytic activity, and the ECAR/OCR ratio were all declined. Same results were observed in the sh1/2-HER2 transfection group in ESCC cells (**Figures 4E** **–I**). By comparison, the effect of FOXM1 and HER2 overexpression on the aerobic glycolysis of ESCC cells was respectively verified in **Supplementary Figures S4B–J**, which further manifested that both FOXM1 and HER2 could effectively promote the aerobic glycolysis of ESCC cells. After that, experimental groups were divided into sh-NC, sh1-FOXM1, sh1-FOXM1+pcDNA3.1, and sh1-FOXM1+pcDNA3.1-HER2 for rescue assays. As shown in **Figure 4J**, the glucose consumption of ESCC cells was inhibited by FOXM1 silencing, while such effect was offset by the cotransfection of pcDNA3.1-HER2. Same opposite results were observed in the total lactate protein, relative PK catalytic activity, and the ECAR/OCR ratio of ESCC cells in different transfection groups (**Figures 4K** **–M**). In conclusion, FOXM1 promoted the aerobic glycolysis of ESCC cells *via* upregulating HER2 expression.

### Exosomal miR-620 Is Highly Secreted in ESCC

**Figure 5:**
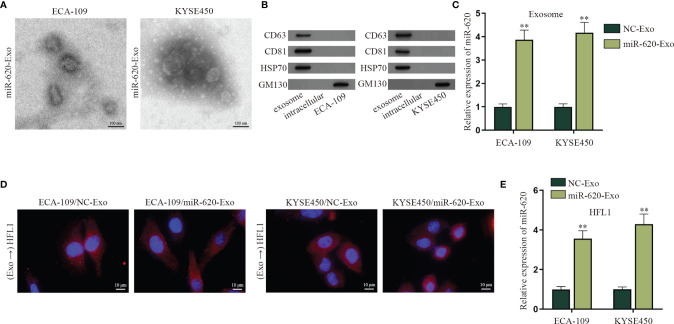
Exosomal miR-620 is highly secreted in ESCC. **(A)** The morphology of exosomes secreted from miR-620-overexpressed ESCC cells was observed through electron microscopy. **(B)** Western blot was used to detect the protein expression of exosome-specific marker proteins (CD63, CD81, and HSP70) and the negative marker GM130 in ESCC cells receiving miR-620-Exo. **(C)** The expression of miR-620 in NC-Exo and miR-620-Exo groups was detected by qRT-PCR. **(D)** Fluorescence microscope was applied to observe the fluorescence of lung fibroblast cells (HFL1) receiving ECA-109/miR-620-Exo and KYSE450/miR-620-Exo. **(E)** The expression of miR-620 in HFL1 cells receiving miR-620-Exo was detected by qRT-PCR. ^**^ *p* < 0.01.

According to the qRT-PCR data in **Fig. S5A**, the expression level of miR-620 in the supernatant of ESCC cells was lower than that of normal Het-1A cell, whereas the intracellular expression of miR-620 in ESCC cells was higher than that that in normal Het-1A cell. Previous study has reported that miRNAs can be secreted to the extracellular environment by membrane-enclosed vesicles (such as exosomes) (6). Therefore, we tried to explore whether miR-620 may play its regulatory role through exosomes. At first, we observed through electron microscope that the exosomes secreted from miR-620-overexpressed ESCC cells (named miR-620-Exo) was 40–100 nm in diameter with oval-shaped vesicles, which conformed to the basic features of exosomes (**Figure 5A**; **Supplementary Figure S5B**). Meanwhile, the expression of exosome-specific marker proteins (CD63, CD81, and HSP70) was detected in ESCC cell-derived exosomes instead of the exosome-negative marker GM130 (**Figure 5B**), and qRT-PCR data showed that miR-620 was highly expressed in the miR-620-Exo group (**Figure 5C**). In order to examine whether the receptor cells could accept the exosomes secreted from the donor cells as well as the exosomal miR-620, we constructed Cy3-miR-620 vectors in the exosomes secreted from ESCC cells (ECA-109/NC-Exo and KYSE450/NC-Exo) and the exosomes secreted from miR-620-overexpresed ESCC cells (ECA-109/miR-620-Exo and KYSE450/miR-620-Exo), as previously described (23). During Transwell assays, the donor ESCC cells containing Cy3-miR-620 were cultured for 24 h and then collected and seeded into the apical chamber of Transwell, and the receptor lung fibroblast cells (HFL1) cells were placed into the basolateral chamber. Through fluorescence microscope, it was observed that most of the receptor cells displayed red fluorescence, indicating that miR-620 could be transmitted through exosomes (**Figure 5D**). With the application of PKH67 Green Fluorescent Cell Linker Kit, it was further manifested that PKH67-marked exosomes were absorbed and internalized by ESCC cells (**Supplementary Figure S5C**), and miR-620-Exo could enhance the expression of miR-620 in the receptor HFL1 cells (**Figure 5E**). Moreover, it was shown that miR-620 expression remained basically unchanged compared with the control group after we treated exosomes with RNase A, while the addition of Triton X-100 declined miR-620 expression in the exosomes, which proved that miR-620 was located in the membrane (**Supplementary Figure S5D**).

### Exosomal miR-620 Inhibits the Aerobic Glycolysis of Lung Fibroblasts

**Figure 6:**
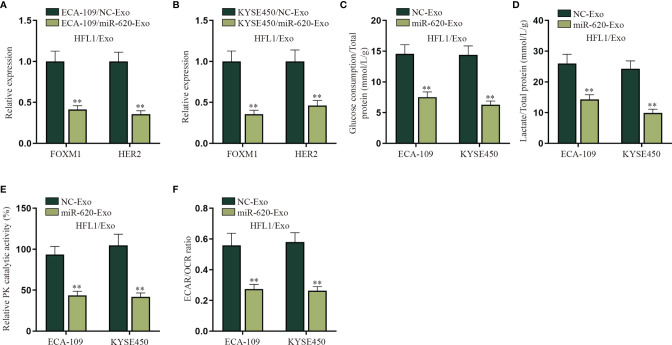
Exosomal miR-620 inhibits the aerobic glycolysis of lung fibroblasts. **(A, B)** qRT-PCR was used to detect the expression of FOXM1 and HER2 in HFL1/Exo cells receiving ECA-109/miR-620-Exo and KYSE450/miR-620-Exo. **(C–F)** qRT-PCR was utilized to detect the glucose consumption, total lactate protein, relative PK catalytic activity, and the ECAR/OCR ratio in HFL1/Exo cells accepting different cancer-secreted exosomal miR-620. ^**^ *p* < 0.01.

An increasing number of evidence has proven that miRNA can regulate cell function by transferring exosomes to adjacent or distant cells (7, 8). For example, it has been reported that breast cancer-secreted miR-122 reprograms glucose metabolism to promote metastasis (20); meanwhile, it has been illustrated that cancer cell-secreted IGF2 instigates fibroblasts and bone marrow-derived vascular progenitor cells to promote cancer progression (21). Therefore, we wanted to explore whether exosomal miR-620 may regulate the aerobic glycolysis of lung fibroblast cells (HFL1). HFL1 cells absorbing the exosomes were renamed HFL1/Exo, and then it was discovered from qRT-PCR that the expression of FOXM1 and HER2 was decreased in HFL1 cells receiving ECA-109/miR-620-Exo and KYSE450/miR-620-Exo (**Figures 6A, B** **)**. In addition, it was observed in **Figures 6C** **–F** that the glucose consumption, total lactate protein, relative PK catalytic activity, and the ECAR/OCR ratio were all reduced in HFL1 cells receiving miR-620-Exo, which indicated that exosomal miR-620 inhibited the aerobic glycolysis of HFL1 cells.

### Exosomal miR-620 Inhibits the Aerobic Glycolysis of Mouse Lung Fibroblasts to Promote ESCC Metastasis

**Figure 7:**
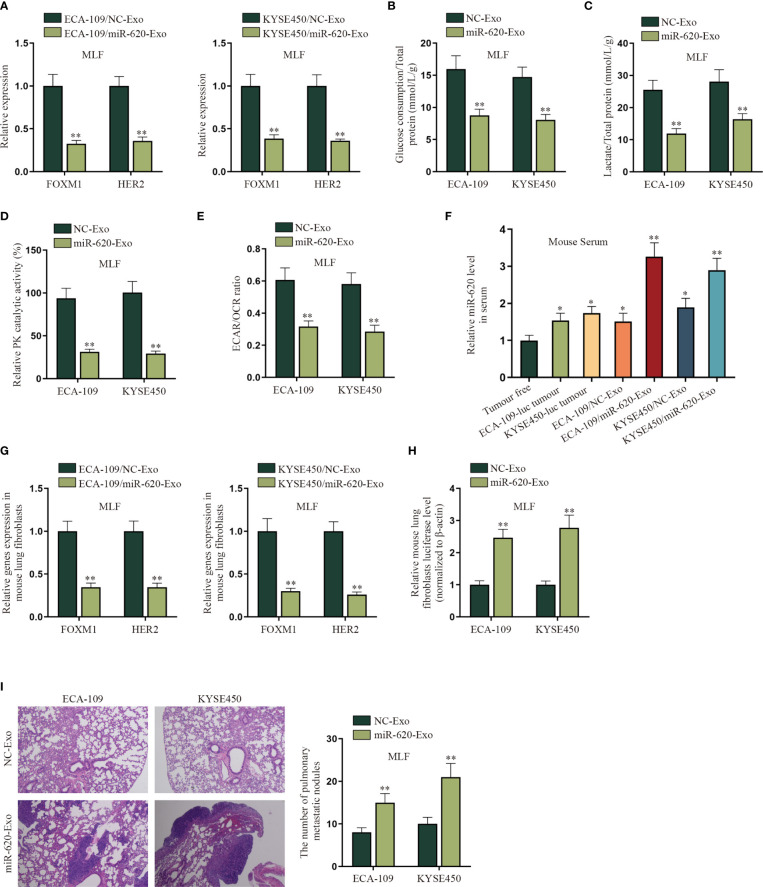
Exosomal miR-620 inhibits the aerobic glycolysis of lung fibroblasts and promotes ESCC metastasis. **(A)** The expression of FOXM1 and HER2 in MLF cells accepting different cancer-secreted exosomal miR-620 was detected by qRT-PCR. **(B–E)** qRT-PCR was utilized to detect the glucose consumption, total lactate protein, relative PK catalytic activity, and the ECAR/OCR ratio in MLF accepting different cancer-secreted exosomal miR-620. **(F)** Luciferase-labeled ESCC cells (ECA-109-luc and KYSE450-luc) were constructed, and qRT-PCR was used to assess the expression level of miR-620 in mouse serum in ESCC cells accepting exosomal miR-620 (ECA-109/miR-620-Exo and KYSE450/miR-620-Exo) in comparison with tumor-transplanted groups (ECA-109-luc tumor and KYSE450-luc tumor). **(G)** qRT-PCR was used to detect the expression of FOXM1 and HER2 in MLF treated with ECA-109/miR-620-Exo and KYSE450/miR-620-Exo. **(H)** qRT-PCR was used to detect the relative MLF luciferase level in mice treated with miR-620-Exo. **(I)** The metastasis of mice was observed through HE staining, and the number of pulmonary metastatic nodules was measured in different groups. ^*^ *p* < 0.05, ^**^ *p* < 0.01.

In order to verify whether ESCC cell-secreted miR-620 may regulate the aerobic glycolysis of premetastatic mouse lung fibroblasts (MLF), related *in vivo* were conducted. qRT-PCR results show that FOXM1 and HER2 expression was reduced in MLF cells receiving ECA-109/miR-620-Exo and KYSE450/miR-620-Exo (**Figure 7A**). Moreover, it was discovered that after MLF received exosomal miR-620, the glucose consumption, total lactate protein, relative PK catalytic activity, as well as the ECAR/OCR ratio were all decreased (**Figures 7B** **–E**), indicating that exosomal miR-620 could inhibit the aerobic glycolysis of MLF.

Furthermore, we investigated the effect of exosomal miR-620 on ESCC metastasis. First of all, the mice were pretreated with exosomes secreted from different ESCC cells for 6 weeks. Luciferase-labeled ESCC cells (ECA-109-luc and KYSE450-luc) were then injected intracardiac, with exosome treatment for another 3 weeks. As shown on **Figure 7F**, qRT-PCR data manifested that the expression level of miR-620 in mouse serum was higher in ESCC cells accepting exosomal miR-620 (ECA-109/miR-620-Exo and KYSE450/miR-620-Exo) than in tumor-transplanted groups (ECA-109-luc tumor and KYSE450-luc tumor). Subsequently, it was analyzed that the expression of FOXM1 and HER2 was reduced in MLF receiving ECA-109/miR-620-Exo and KYSE450/miR-620-Exo (**Figure 7G**). Moreover, it was found that the relative MLF luciferase level was higher in mice treated with miR-620-Exo, which indicated that ESCC metastasis was more significant in mice with higher miR-620 expression in serum (**Figure 7H**). The number of pulmonary metastatic nodules was further tested to be enhanced in MLF receiving ECA-109/miR-620-Exo and KYSE450/miR-620-Exo (**Figure 7I**). To conclude, exosomal miR-620 inhibited the aerobic glycolysis of MLF and thus promoted ESCC metastasis.

### MiR-620 Inhibits the Aerobic Glycolysis of Primary Tumors and Promotes ESCC Metastasis

**Figure 8:**
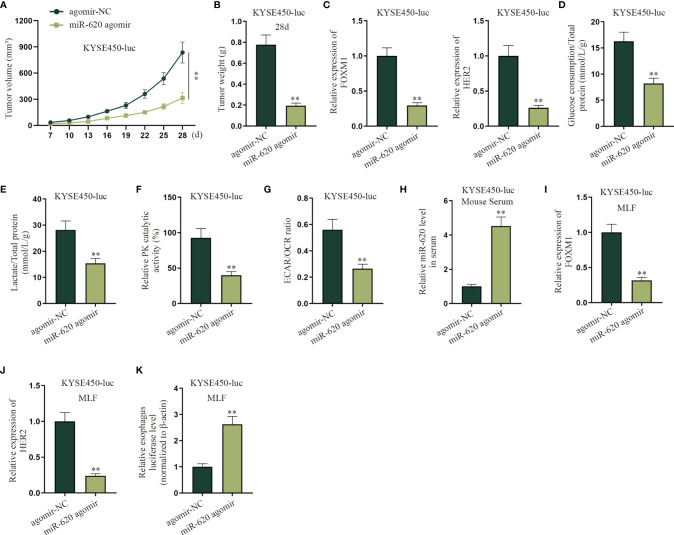
MiR-620 inhibits the aerobic glycolysis of primary tumors and promotes ESCC metastasis. **(A, B)** Orthotopic mammary xenografts were constructed in mice, and the tumor volume as well as tumor weight of tumors were examined *via* qRT-PCR after miR-620 was overexpressed. **(C)** qRT-PCR was utilized to detect the expression of FOXM1 and HER2 in KYSE450-luc cells upon miR-620 overexpression. **(D–G)** qRT-PCR was utilized to test the glucose consumption, total lactate protein, relative PK catalytic activity, and the ECAR/OCR ratio in miR-620-overexpressed KYSE450-luc cells. **(H)** The expression of miR-620 in serum was measured by qRT-PCR in miR-620-overexpressed KYSE450-luc mouse serum. **(I, J)** The expression of FOXM1 and HER2 in MLF upon miR-620 overexpression was detected by qRT-PCR. **(K)** qRT-PCR was utilized to measure the relative MLF luciferase level in the xenograft tumors treated with enhanced miR-620 expression. ^**^ *p* < 0.01.

To determine if the primary tumor-secreted miR-620 enhances metastasis, orthotopic mammary xenografts were constructed. It was shown that after miR-620 was upregulated in KYSE450-luc cells, the xenograft tumors displayed a slower growth tendency as well as a lighter tumor weight at the 28th day (**Figures 8A, B** **)**. It was then observed through qRT-PCR that the expression of FOXM1 and HER2 in KYSE450-luc cells was reduced upon miR-620 overexpression (**Figure 8C**). In addition, the aerobic glycolysis of KYSE450-luc cells was discovered to be suppressed by miR-620 overexpression, as the glucose consumption, total lactate protein, relative PK catalytic activity, and the ECAR/OCR ratio of KYSE450-luc cells were all declined (**Figures 8D** **–G**). To identify the effect of miR-620 on the ESCC metastasis, miR-620 was overexpressed in KYSE450-luc cells, which turned out that miR-620 expression was enhanced in mouse serum (**Figure 8H**). It was then observed that the expression of FOXM1 and HER2 in KYSE450-luc cells was reduced in MLF after miR-620 was overexpressed (**Figures 8I, J** **)**, and the relative MLF luciferase level was higher in the xenograft tumors treated with enhanced miR-620 expression (**Figure 8K**). The above findings showed that miR-620 inhibited the aerobic glycolysis of primary tumors while promoting ESCC metastasis.

## Discussion

ESCC is a common malignant tumor of the digestive system worldwide, especially in China. It is featured by advanced diagnosis and poor prognosis as a result of limited and ineffective early detection methods at an early stage (25). Through the GEO database (id: GSE122497), we found miR-620 in the serum miRNAs with obviously high expression. As miR-620 has been reported to exert an important role in many cancers (26, 27) but not in ESCC, we chose to verify its upregulation pattern in ESCC cells. In line with functional assays, miR-620 was verified to be an oncogene that inhibits cell proliferation, migration, invasion, and EMT process in ESCC.

Energy metabolism reprogramming is an emerging hotspot in cancer research. Altered aerobic glycolysis represents a well-recognized characteristic of cancer cell energy metabolism, termed as the Warburg effect. Even in the presence of abundant oxygen, a majority of tumor cells produce substantial amounts of energy through a high glycolytic metabolism (28). It has been documented that cancer-secreted miRNA can reprogram aerobic glycolysis of lung fibroblasts to promote metastasis in breast cancer (20), but such study has not been conducted in ESCC. In this study, we verified that miR-620 overexpression and the addition of 2-DG exerted consistent outcomes on ESCC cells, suggesting that miR-620 could regulate aerobic glycolysis in ESCC. Next, we further explored the specific molecular mechanisms of miR-620 in the regulation of aerobic glycolysis. Through bioinformatics prediction, FOXM1 was selected and verified to be the downstream target of miR-620, and miR-620 could bind to FOXM1 3′UTR to reduce FOXM1 mRNA stability. In addition, FOXM1 was verified to bind to the HER2 promoter and transcriptionally activated HER2 in ESCC. Through a series of rescue assays, it was proved that FOXM1 could regulate aerobic glycolysis in ESCC through HER2. The above data suggested the miR-620/FOXM1/HER2 axis in regulating the process of aerobic glycolysis.

It has been reported that miRNAs can be secreted into the extracellular environment by extracellular vesicles, such as exosomes (6). Exosomes are effective carriers for the intercellular material transfer of material (29), and the function of exosomal miRNAs as well as miRNA dysregulation in cancer-associated fibroblasts has been reported (19). In this study, through exosome extraction and uptake experiments, we discovered that exosomal miR-620 was highly secreted in ESCC cells. Also, it was found that human embryonic lung fibroblast HFL1 cells could absorb and internalize exosomes, which was renamed as HFL1/Exo for the follow-up studies.

More and more evidences have demonstrated that miRNAs can regulate cell function by transferring exosomes to the neighboring or distant cells (7, 8). It has been reported that breast cancer-secreted miR-122 reprograms lung fibroblast aerobic glycolysis and thus promotes metastasis (20), and it was discovered that cancer cell-secreted IGF2 instigates fibroblasts and bone marrow-derived vascular progenitor cells to promote cancer progression (21). In this study, we verified through mechanism assays that exosomal miR-620 downregulated aerobic glycolysis of HFL1. Finally, *in vivo* experiments were carried out and demonstrated that ESCC-secreted miR-620 could promote reprogram aerobic glycolysis in lung fibroblasts and thus promote metastasis in ESCC. A graphical abstract has been provided for better understanding.

In summary, our study suggests that exosomal miR-620 secreted by ESCC cells regulates HFL1 aerobic glycolysis *via* FOXM1/HER2 signaling and promotes ESCC metastasis by reprogramming the aerobic glycolysis of lung fibroblasts.

Our study has some limitations; for example, we chose to study miR-620 at the beginning of our study and then found through experiments that it may exert functions through exosomes. There are many other potential miRNAs in the exosomes of ESCC, but we did not conduct related sequencing for them to better select the target miRNA, which needs further sequencing test in the future to help us complete our research. In addition, human epidermal growth factor receptor 2-positive (HER2+) breast cancer makes up about 20% of all invasive breast cancers, and Herceptin (trastuzumab), known as a human monoclonal antibody that interferes with the HER2 receptor, is currently the only FDA-approved therapeutic antibody for HER2-positive breast cancer (30) (31). In our study, HER2 was identified to be the target of FOXM1, which further participated in the miR-620/FOXM1/HER2 axis to regulate ESCC aerobic glycolysis. As the ESCC cell lines used in our study did not involve HER2 positivity, we did not focus on exploring the potential mechanism of Herceptin here. On the other hand, as we utilized lung fibroblasts in mechanism assays, we will try to utilize lung fibroblasts of esophageal origin to further complete our investigations on the aerobic glycolysis of ESCC. The extraction of exosomes using ultracentrifugation is controversial as it cannot exclude the contamination of other particles (such as microvesicles), while there are also reports proving that exosomes were extracted through this way (32) (33). We will consider this important matter and try to apply other approaches to extract exosomes in future researches. All in all, we hope that this study may provide a novel insight into therapeutic options for future ESCC treatment.

## Data Availability Statement

The original contributions presented in the study are included in the article/**Supplementary Material**. Further inquiries can be directed to the corresponding author.

## Ethics Statement

All the relevant animal experiments were carried out in accordance with the Animal Care and Use Committee guidelines of the First Affiliated Hospital of Soochow University. Written informed consent was obtained from the owners for the participation of their animals in this study.

## Author Contributions

FL and YZ conceived and designed the study. YW and HL performed the experiments. SL and BP analyzed the data. LS and YX prepared all the figures. DJ wrote the paper. All authors read and approved the final manuscript.

## Funding

This work was supported by Suzhou Health Talent Training Project (GSWS2020008) and the National Natural Science Foundation of China (81972800).

## Conflict of Interest

The authors declare that the research was conducted in the absence of any commercial or financial relationships that could be construed as a potential conflict of interest.

## Publisher’s Note

All claims expressed in this article are solely those of the authors and do not necessarily represent those of their affiliated organizations, or those of the publisher, the editors and the reviewers. Any product that may be evaluated in this article, or claim that may be made by its manufacturer, is not guaranteed or endorsed by the publisher.

## Supplementary Material

Supplementary Figure 1 MiR-620 regulates the phenotype of ESCC cells. **(A)** With the application of GEO database (GSE122497), miR-620 was selected through large-scale serum microRNA profiling as a significantly up-regulated miRNA. **(B)** CCK-8 assay was carried out to assess the effect of miR-620 silencing on the proliferation of ESCC cells. **(C)** Wounding healing assay was conducted to evaluate the migration of ESCC cells upon miR-620 silencing. **(D)** The migration as well as invasion of ESCC cells transfected with miR-620 inhibitor was evaluated through Transwell assay. **(E)** The phenotype of ESCC cells upon miR-620 knockdown was observed through microscope. **(F)** Western blot was utilized to detect the expression of the EMT-related proteins (E-cadherin and Vimentin) upon miR-620 knockdown. **P < 0.01.

Supplementary Figure 2 MiR-620 regulates the aerobic glycolysis of ESCC cells. **(A)** Glucose determination kit was applied to examine the glucose determination of miR-620 inhibitor-transfected ESCC cells. **(B)** Lactic acid determination kit was used to detect lactic acid content in ESCC cells upon miR-620 silencing. **(C)** Pyruvate kinase activity assay kit was applied to measure the PK content in miR-620 inhibitor-transfected ESCC cells. **(D)** The ECAR/OCR ratio in miR-620 inhibitor-transfected ESCC cells was tested by Seahorese XF Extracellular Flux Analyzer. **(E–H)**. ESCC cells were treated with 50 or 100μM 2-DG (the glycolytic inhibitor) for 48h, and the glucose consumption, total lactate protein, relative PK catalytic activity and the ECAR/OCR ratio were respectively examined *via* qRT-PCR in different groups (inhibitor-NC, miR-620 inhibitor, miR-620 inhibitor+NC and miR-620 inhibitor+2-DG). **P < 0.01.

Supplementary Figure 3 MiR-620 regulates HER2 expression through FOXM1. **(A, B)**. Through GEO database (GSE75241), the top 30 genes up-regulated in ESCC were selected, and potential mRNAs which could bind to miR-620 were selected through starBase database. **(C)** The expression of 8 candidates (ITM2C, FNDC38, CD276, LAMC2, LHFPL2, LAMC1, PARVB and FOXM1) was measured by qRT-PCR in ESCC cells transfected with miR-620 mimics. **(D)** KEGG database was applied to forecast potential genes related to aerobic glycolysis. E. HER2 was forecast to be the target gene of FOXM1 through hTFtarget database. **P < 0.01.

Supplementary Figure 4 FOXM1 regulates the aerobic glycolysis of ESCC cells through HER2. **(A)** FOXM1 expression was enhanced in ESCC cells by the transfection of pcDNA3.1-FOXM1. **(B–E)**. qRT-PCR was utilized to test the glucose consumption, total lactate protein, relative PK catalytic activity and the ECAR/OCR ratio in FOXM1-overexpressed ESCC cells. **(F–J)**. HER2 expression was enhanced in ESCC cells, and then qRT-PCR was utilized to test the glucose consumption, total lactate protein, relative PK catalytic activity and the ECAR/OCR ratio in HER2-overexpressed ESCC cells. **P < 0.01.

Supplementary Figure 5 Exosomal miR-620 is highly secreted in ESCC cells. **(A)** The expression of miR-620 in supernatant of normal esophageal cell line Het-1A and ESCC cell lines was detected by qRT-PCR. **(B)** The morphology of exosomes secreted from ECA-109 and KYSE450 (exosomes labeled NC-Exo) were observed through electron microscopy. **(C)** PKH67 staining-laser confocal microscopy was used to observe whether exosomes could enter recipient cells. **(D)** RNase A, along with Triton X-100 was used to treat the culture medium of ESCC cells and the expression of miR-620 in different groups was measured. **P < 0.01.

Supplementary Table 1 Information on related sequences.
