# Supplementary figures and images for: Cancer-Secreted Exosomal MiR-620 Inhibits ESCC Aerobic Glycolysis via FOXM1/HER2 Pathway and Promotes Metastasis
Source: Front Oncol. 2022 May 16;12:756109. doi: 10.3389/fonc.2022.756109 (PMC9148961; doi:10.3389/fonc.2022.756109)

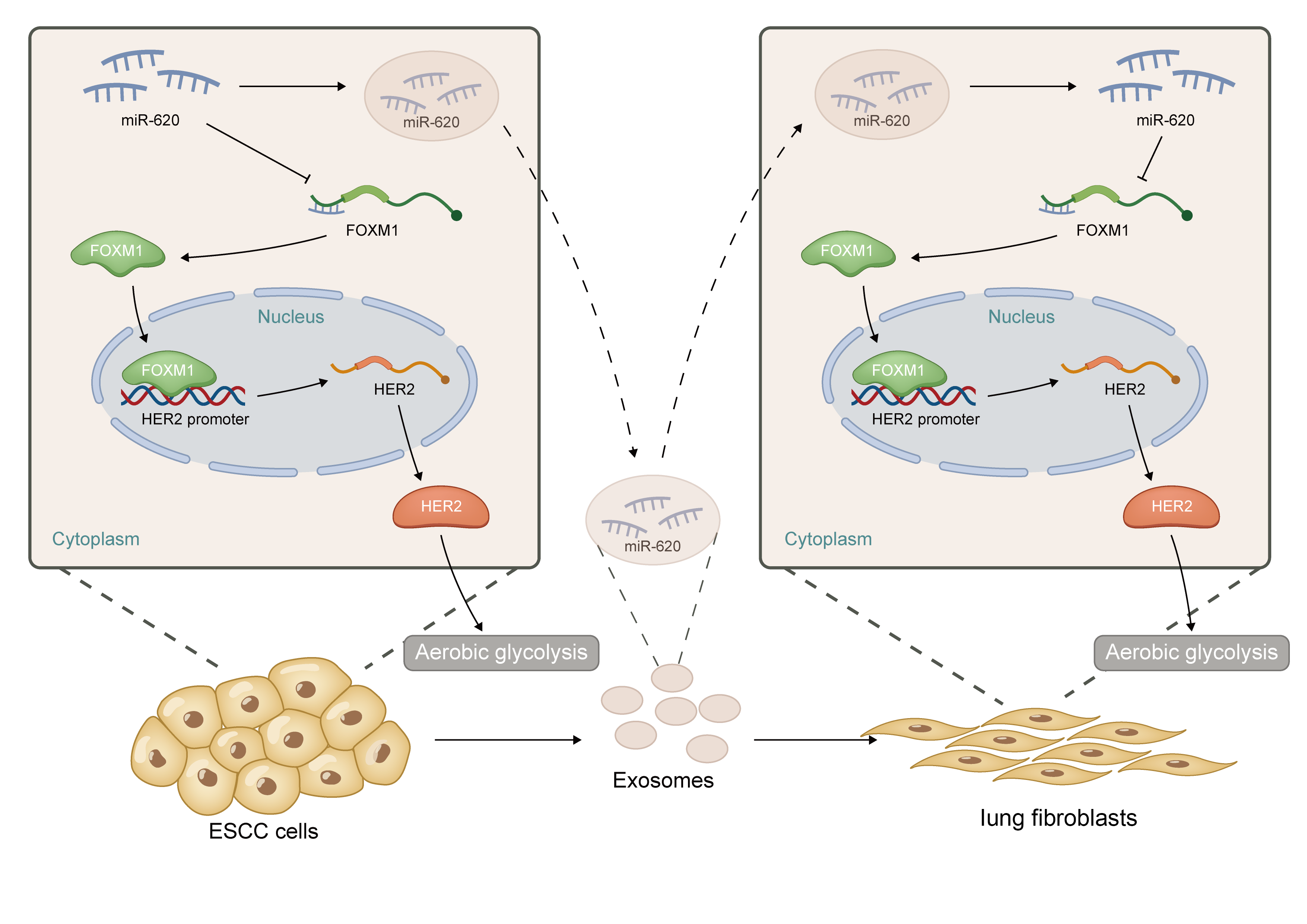

Supplement: Supplementary Figure 1 — MiR-620 regulates the phenotype of ESCC cells. (A) With the application of GEO database (GSE122497), miR-620 was selected through large-scale serum microRNA profiling as a significantly up-regulated miRNA. (B) CCK-8 assay was carried out to assess the effect of miR-620 silencing on the proliferation of ESCC cells. (C) Wounding healing assay was conducted to evaluate the migration of ESCC cells upon miR-620 silencing. (D) The migration as well as invasion of ESCC cells transfected with miR-620 inhibitor was evaluated through Transwell assay. (E) The phenotype of ESCC cells upon miR-620 knockdown was observed through microscope. (F) Western blot was utilized to detect the expression of the EMT-related proteins (E-cadherin and Vimentin) upon miR-620 knockdown. **P < 0.01. [file Image_1.tif]

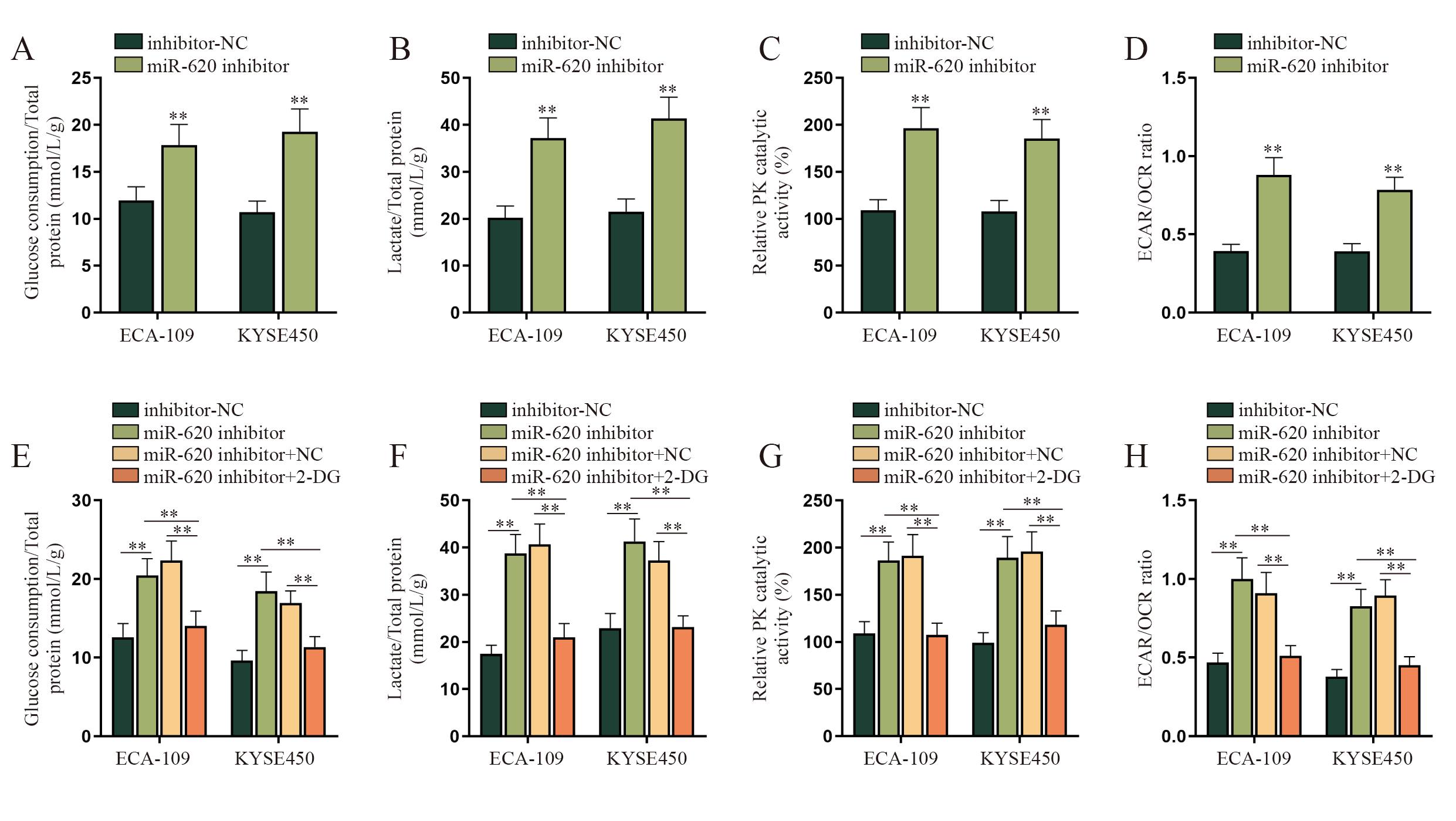

Supplement: Supplementary Figure 2 — MiR-620 regulates the aerobic glycolysis of ESCC cells. (A) Glucose determination kit was applied to examine the glucose determination of miR-620 inhibitor-transfected ESCC cells. (B) Lactic acid determination kit was used to detect lactic acid content in ESCC cells upon miR-620 silencing. (C) Pyruvate kinase activity assay kit was applied to measure the PK content in miR-620 inhibitor-transfected ESCC cells. (D) The ECAR/OCR ratio in miR-620 inhibitor-transfected ESCC cells was tested by Seahorese XF Extracellular Flux Analyzer. (E–H). ESCC cells were treated with 50 or 100μM 2-DG (the glycolytic inhibitor) for 48h, and the glucose consumption, total lactate protein, relative PK catalytic activity and the ECAR/OCR ratio were respectively examined via qRT-PCR in different groups (inhibitor-NC, miR-620 inhibitor, miR-620 inhibitor+NC and miR-620 inhibitor+2-DG). **P < 0.01. [file Image_2.tif]

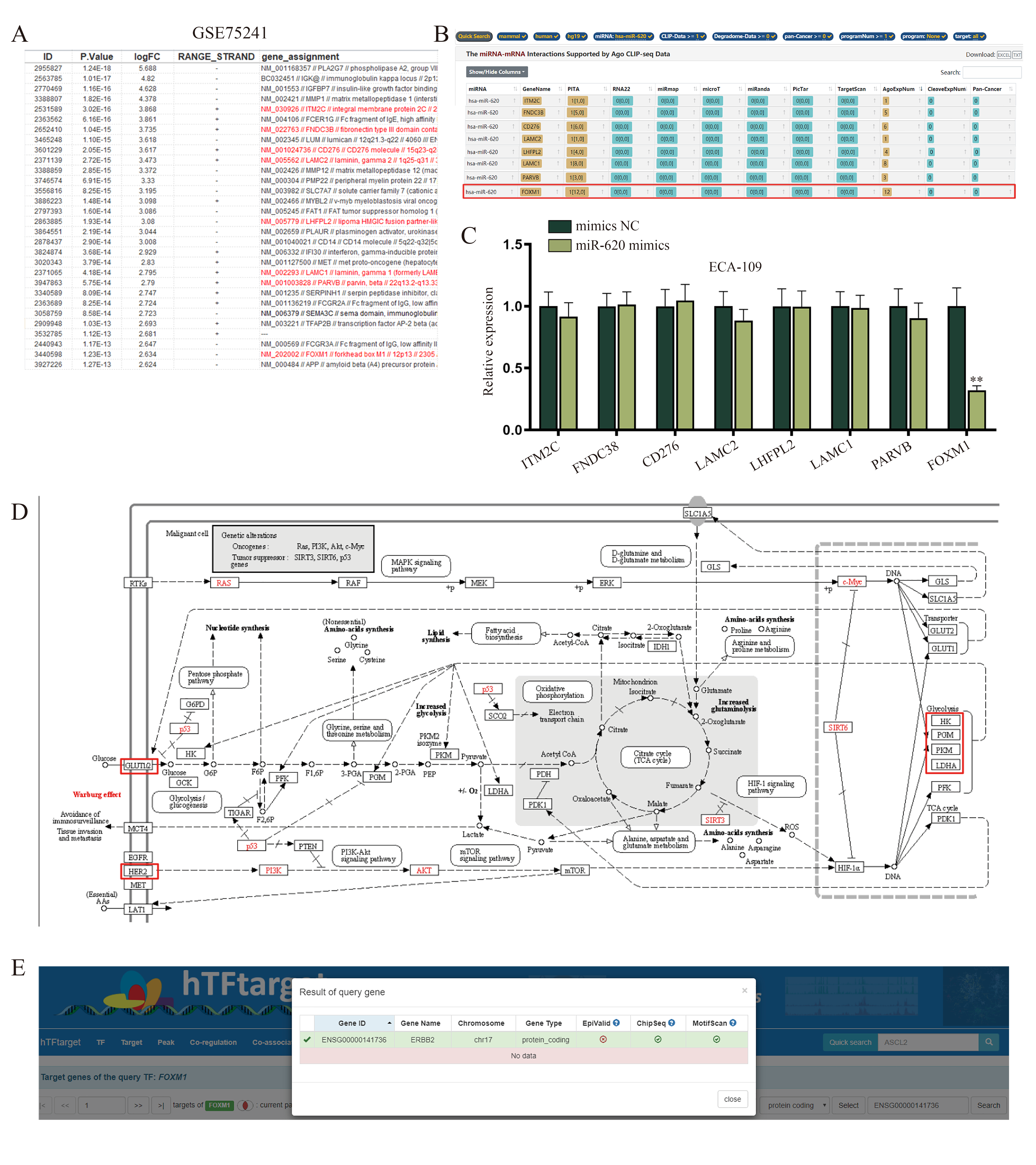

Supplement: Supplementary Figure 3 — MiR-620 regulates HER2 expression through FOXM1. (A, B). Through GEO database (GSE75241), the top 30 genes up-regulated in ESCC were selected, and potential mRNAs which could bind to miR-620 were selected through starBase database. (C) The expression of 8 candidates (ITM2C, FNDC38, CD276, LAMC2, LHFPL2, LAMC1, PARVB and FOXM1) was measured by qRT-PCR in ESCC cells transfected with miR-620 mimics. (D) KEGG database was applied to forecast potential genes related to aerobic glycolysis. E. HER2 was forecast to be the target gene of FOXM1 through hTFtarget database. **P < 0.01. [file Image_3.tif]

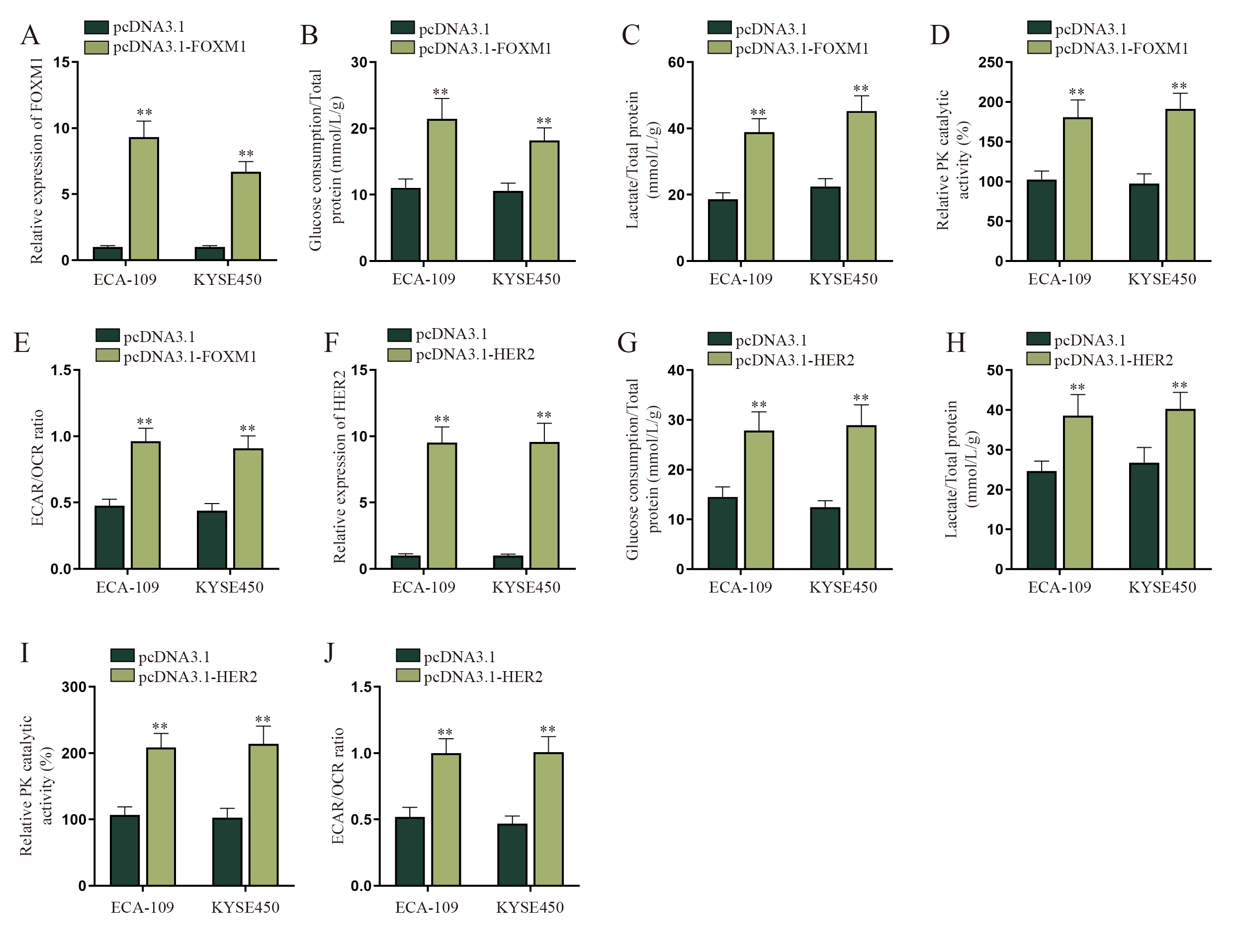

Supplement: Supplementary Figure 4 — FOXM1 regulates the aerobic glycolysis of ESCC cells through HER2. (A) FOXM1 expression was enhanced in ESCC cells by the transfection of pcDNA3.1-FOXM1. (B–E). qRT-PCR was utilized to test the glucose consumption, total lactate protein, relative PK catalytic activity and the ECAR/OCR ratio in FOXM1-overexpressed ESCC cells. (F–J). HER2 expression was enhanced in ESCC cells, and then qRT-PCR was utilized to test the glucose consumption, total lactate protein, relative PK catalytic activity and the ECAR/OCR ratio in HER2-overexpressed ESCC cells. **P < 0.01. [file Image_4.tif]

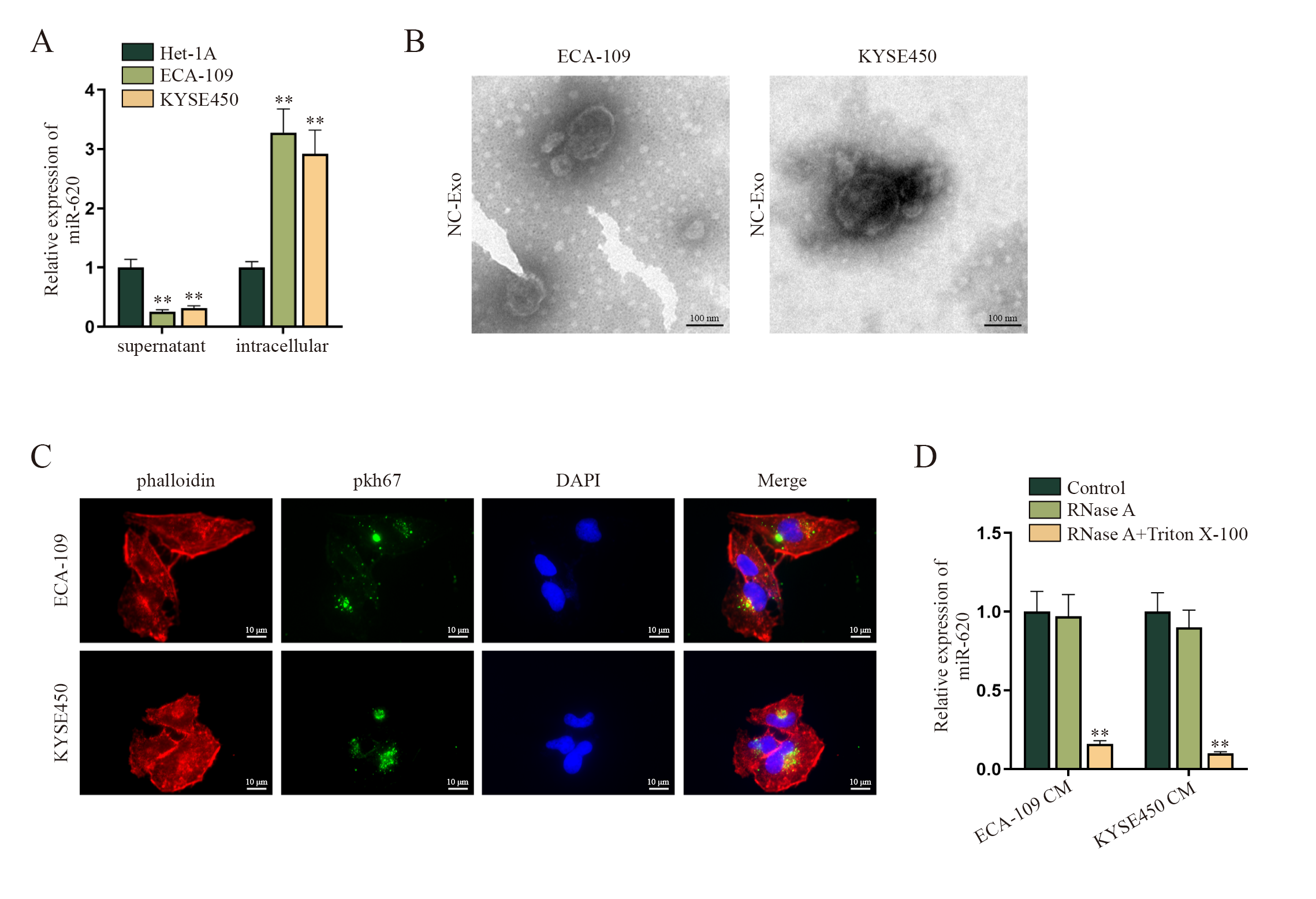

Supplement: Supplementary Figure 5 — Exosomal miR-620 is highly secreted in ESCC cells. (A) The expression of miR-620 in supernatant of normal esophageal cell line Het-1A and ESCC cell lines was detected by qRT-PCR. (B) The morphology of exosomes secreted from ECA-109 and KYSE450 (exosomes labeled NC-Exo) were observed through electron microscopy. (C) PKH67 staining-laser confocal microscopy was used to observe whether exosomes could enter recipient cells. (D) RNase A, along with Triton X-100 was used to treat the culture medium of ESCC cells and the expression of miR-620 in different groups was measured. **P < 0.01. [file Image_5.tif]
